# Role of Nutrition in Alcoholic Liver Disease: *Summary of the Symposium at the ESBRA 2017 Congress*

**DOI:** 10.3390/biom8020016

**Published:** 2018-03-26

**Authors:** Kusum K. Kharbanda, Martin J. J. Ronis, Colin T. Shearn, Dennis R. Petersen, Samir Zakhari, Dennis R. Warner, Ariel E. Feldstein, Craig J. McClain, Irina A. Kirpich

**Affiliations:** 1 Research Service, Veterans Affairs Nebraska-Western Iowa Health Care System, Omaha, NE 68105, USA; kkharbanda@unmc.edu; 2 Department of Internal Medicine, University of Nebraska Medical Center, Omaha, NE 68105, USA; 3 Department of Biochemistry and Molecular Biology, University of Nebraska Medical Center, Omaha, NE 68105, USA; 4 Department of Pharmacology and Experimental Therapeutics, Louisiana State University Health Sciences Center, New Orleans, LA 70112, USA; mronis@lsuhsc.edu; 5 Department of Pharmaceutical Sciences, University of Colorado Anschutz Medical Campus, Denver, CO 80045, USA; colin.shearn@ucdenver.edu (C.T.S.); dennis.petersen@ucdenver.edu (D.R.P.); 6 Distilled Spirits Council, Washington, DC 20005, USA; samir.zakhari@distilledspirits.org; 7 Division of Gastroenterology, Hepatology, and Nutrition, Department of Medicine, University of Louisville, Louisville, KY 40202, USA; dennis.warner@louisville.edu (D.R.W.); craig.mcclain@louisville.edu (C.J.M.); 8 Division of Gastroenterology, Department of Pediatrics, University of California, San Diego, CA 92037, USA; afeldstein@ucsd.edu; 9 Department of Pharmacology and Toxicology, University of Louisville, Louisville, KY 402202, USA;; 10 University of Louisville Alcohol Center, University of Louisville School of Medicine, Louisville, KY 40202, USA; 11 Robley Rex Veterans Medical Center, Louisville, KY 40202, USA; 12 Hepatobiology and Toxicology Program, University of Louisville, Louisville, KY 402202, USA

**Keywords:** alcoholic liver disease, nutrition, fat, carbohydrates, betaine, malnutrition, zinc, nutritional support in ALD

## Abstract

The symposium, “Role of Nutrition in Alcoholic Liver Disease”, was held at the European Society for Biomedical Research on Alcoholism Congress on 9 October 2017 in Crete, Greece. The goal of the symposium was to highlight recent advances and developments in the field of alcohol and nutrition. The symposium was focused on experimental and clinical aspects in relation to the role of different types of dietary nutrients and malnutrition in the pathogenesis of alcoholic liver disease (ALD). The following is a summary of key research presented at this session. The speakers discussed the role of dietary fats and carbohydrates in the development and progression of alcohol-induced multi-organ pathology in animal models of ALD, analyzed novel nutrition-related therapeutics (specifically, betaine and zinc) in the treatment of ALD, and addressed clinical relevance of malnutrition and nutrition support in ALD. This summary of the symposium will benefit junior and senior faculty currently investigating alcohol-induced organ pathology as well as undergraduate, graduate, and post-graduate students and fellows.

## 1. Introduction

Alcohol-induced liver dysfunction is a significant health problem worldwide for which there is no current food and drug administration (FDA)-approved therapy. Alcoholic liver disease (ALD) refers to a wide histological spectrum of liver pathologies, including steatosis (fatty liver), steatohepatitis (characterized by a combination of hepatic fat accumulation and inflammation), liver fibrosis and cirrhosis. To date, alcohol abstinence is the most effective strategy to prevent, and/or to attenuate the disease. The cross-talk among multiple organs/tissues, e.g., the gut-liver, the gut-liver-brain, and the white adipose tissue (WAT)-liver axes, plays a significant role in the ALD pathogenesis. Overall, oxidative stress and inflammation are key mediators in ALD development [1,2,3]. 

Multiple factors are involved in the development of ALD, including genetic, epigenetic, and environmental factors, such as nutrition. Alcohol and nutrients can interact at multiple levels. Excessive alcohol ingestion alters the metabolism of most nutrients. Alcohol consumption activates enzymatic and non-enzymatic lipid oxidation contributing to hepatic oxidative stress. Ethanol-mediated alterations in methionine metabolism result in reduced levels of antioxidants, *S*-adenosyl-methionine (SAMe) and glutathione, leading to increased oxidative stress and liver injury [4]. Further, heavy alcohol consumption also can cause poor intestinal absorption of certain nutrients (e.g., zinc) or increase nutrient losses. Numerous dietary factors (e.g., Zn, SAMe and betaine supplements, dietary fat enriched in certain fatty acids) have demonstrated beneficial effects in clinical and experimental ALD [5,6,7]. Nutrition plays an important role as supportive therapy [8,9], as nutritional deficiencies commonly occur in patients with ALD, and patients with severe alcoholic hepatitis almost invariably demonstrate some form of malnutrition [10,11]. 

At this symposium, the speakers discussed the role of dietary fats in the development and progression of alcohol-induced liver pathology, focusing specifically on linoleic acid and its oxidized metabolites; discussed the importance of dietary fat/carbohydrate ratio; examined novel nutrition-related therapeutics (specifically, betaine) in the treatment of ALD, and addressed clinical relevance of malnutrition and nutrition support in ALD. All presentations at this symposium supported the notion that nutritional factors play important roles in alcohol-induced multi-organ pathology and could serve as potential preventive/therapeutic targets/options.

## 2. Summary of Presentations at the Symposium

### 2.1. Dietary Linoleic Acid and Its Oxidized Metabolites Exacerbate Liver Injury Caused by Ethanol via Induction of Hepatic Pro-Inflammatory Response

Dennis R. Warner, Ariel E. Feldstein, Craig J. McClain, and Irina A. Kirpich

**Figure 1:**
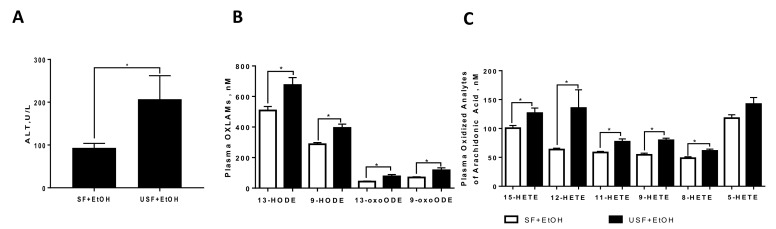
Elevated levels of polyunsaturated fatty acid (PUFA) metabolites in liver injury caused by acute-on-chronic ethanol administration. (**A**) Plasma alanine aminotransferase (ALT) levels were significantly higher in mice fed ethanol and unsaturated fat compared to ethanol and dietary saturated fat; (**B**) oxidized metabolites of linoleic acid; (**C**) oxidized metabolites of arachidonic acid. SF: saturated fat; EtOH: ethanol-fed; USF: unsaturated fat; HODE: hydroxyoctadecadienoic acids; HETE: hydroxyeicosatetraenoic acids; * *p* < 0.05.

Studies from our laboratory and others have demonstrated that dietary fats are important modulators of the toxic effects of ethanol in the liver [12]. It has been previously shown that rodents placed on diets high in unsaturated fat (USF, enriched predominantly in the polyunsaturated fatty acid (PUFA), linoleic acid (LA)) when combined with ethanol showed significantly greater liver injury compared to animals fed ethanol and other types of fat, e.g., saturated fat (SF), enriched in medium chain fatty acids [6,13,14]. This effect may be partially due to the oxidation of LA via the 12/15-lipoxygenase pathway or through non-enzymatic, free radical-mediated oxidation, to generate pro-inflammatory metabolites. In agreement with previously published studies [15,16], we found in two different animal models of ALD (chronic ad-libitum Lieber-deCarli and acute-on-chronic NIAAA models) that in comparison to mice fed SF and ethanol, animals fed USF and ethanol had a greater liver injury which was associated with the increased levels of oxidized LA metabolites (OXLAMs, Figure 1A,B, acute-on-chronic NIAAA model is shown). In addition, we also observed alterations in arachidonic acid (AA) metabolites (e.g., hydroxyeicosatetraenoic acids (HETEs)), many of which are known pro-inflammatory lipid mediators (Figure 1C). For example, 12-HETE induced *Tnf-α*, *Mcp-1*, and *Il-6* expression in macrophages [17,18]. Additionally, an increase in 12-HETE was observed in patients with ALD [19].

To explore the mechanisms by which oxidized linoleic acid metabolites (OXLAMs) may contribute to enhanced liver injury, we tested the hypothesis that ethanol-induced oxidation of LA and the subsequent increase in hepatic and circulating OXLAMs exacerbate liver injury via shifting hepatic macrophages toward the pro-inflammatory (M1) phenotype. To this end, RAW264.7 cells were treated with 5 μM 9- or 13-hydroxyoctadecadienoic acids (HODEs) with or without lipopolysaccharides (LPS) (100 ng/mL). Stimulation of RAW264.7 cells by 9-HODE alone, but not 13-HODE alone, led to a significant increase in *Tnf-α* expression. A similar pattern was observed for the expression of *Mip-2α* and *Mcp-1*, where 9-HODE alone or in combination with LPS enhanced their expression, but 13-HODE alone did not. In contrast, 13-HODE, but not 9-HODE, potentiated LPS-induction of *iNos* expression. These results demonstrate that 9- and 13-HODE have different, and even opposing, roles in regulating cytokine gene expression in RAW264.7 cells, with 9-HODE promoting a pro-inflammatory response (M1 response) to a greater extent than 13-HODE. There was no effect of either 9- or 13-HODE on the expression of M2 macrophage markers (*Arg-1* or *Tgf-β1*, anti-inflammatory response markers), suggesting that 9-HODE was primarily pro-inflammatory and that 13-HODE was mainly neutral or had some anti-inflammatory activity. There are three known receptors for HODES: GPR132, TRPV1, and PPARγ. GPR132 binds to 9-HODE but binds only very weakly to 13-HODE [20]. TRPV1 and PPARγ can bind to both 9- and 13-HODE [21,22]. RAW264.7 cells do not express *Trpv1* [23] and express only very low levels of *Pparγ* [24]. Therefore, the primary response to HODEs in RAW264.7 cells is likely mediated by GPR132 and explains the relative inactivity of 13-HODE in these cells. Future studies on HODE/receptor interactions are needed. 

In summary, the results of the study support the concept that dietary LA, a ω-6-PUFA, exacerbates ethanol-induced liver injury and provides evidence that the increase in OXLAM production and promotion of an OXLAM-mediated pro-inflammatory response might be one of the underlying mechanisms. Of note, there is scant evidence regarding the potential role of metabolites generated from LA and other PUFAs through other metabolic pathways (e.g., the cytochrome p450/epoxide hydrolase pathway), as well as lipid mediators derived from ω-3 PUFAs, such as α-linolenic acid, eicosapentaenoic and docosahexaenoic acids. Given that the majority of PUFA metabolites are potent endogenous signaling molecules that function through multiple pathways, identification of changes in specific lipid mediators might shed new light into the mechanisms contributing to ALD pathogenesis and may reveal novel therapeutic targets and biomarkers of this disease. Further research is required to elucidate the specific role and mechanisms by which each PUFA-derived metabolite exerts its effect during ALD pathogenesis. 

### 2.2. Role of Fat/Carbohydrate Ratio and Dietary Fat Type in Development of Alcoholic Liver Pathology

Martin J.J. Ronis, Colin T. Shearn, and Dennis R. Petersen

**Figure 2:**
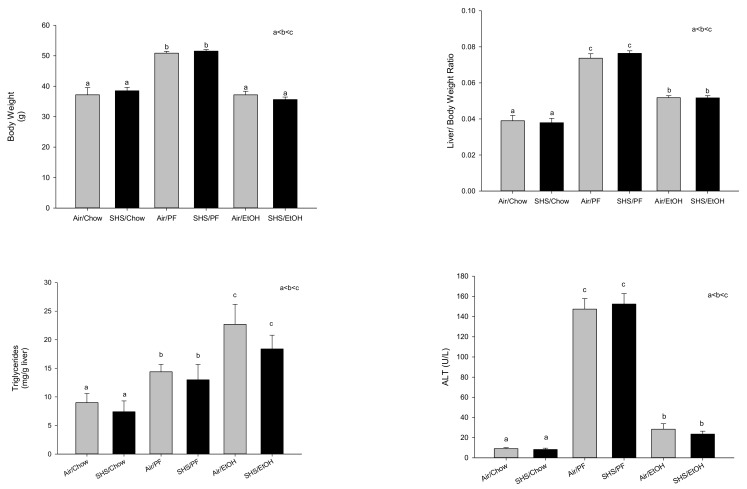
Effects of gestational second-hand smoke (SHS) on body weight and liver pathology in male C57BL/6 mice fed chow or pair-fed Lieber DeCarli liquid diets at up to 28% ethanol calories for 16 weeks beginning on post-natal day 65. PF: pair-fed dextrose/maltodextran. a < b < c, significantly different at *p* < 0.05.

The development of liver pathology following alcohol consumption in experimental animals is highly dependent on dietary macromolecule composition. Rats were fed ethanol at 12–13 g/kg/day intragastrically via isocaloric liquid diets at a level of 187 kcal/kg/day. Ethanol diets high in simple carbohydrates (16% protein, 79% dextrose/maltodextrin, 5% corn oil) or polyunsaturated fats (16% protein, 39% dextrose/maltodextrin, 45% corn oil) were both observed to produce hepatic steatosis. However, this occurred much more rapidly in rats fed ethanol as part of the high carbohydrate diet, within 14 days of feeding, coincident with increased fatty acid synthesis and increased nuclear expression of the carbohydrate response element binding protein (ChREBP) [25]. In contrast to data from other laboratories [26], development of steatosis was not accompanied by any significant effects on serum concentrations of adiponectin, hepatic expression of the histone deacetylase, Sirt-1, or nuclear expression of the steroid regulatory element binding protein, SREBP-1c [25]. Chronic feeding of high carbohydrate control diets in the absence of ethanol for 65 days resulted in development of identical steatosis and liver injury to that seen in the ethanol-high carbohydrate diets [25]. These data are consistent with recent studies from David Crabb’s laboratory [27] and suggest that ethanol is treated metabolically like a carbohydrate. The data also raise an important issue regarding use of dextrose/maltodextran to pair-feed control groups in chronic studies of alcoholic liver injury. Such “control” liquid diets, high in fat and simple carbohydrates are not benign. This is illustrated by the results of a recent chronic feeding study from our laboratory in which we examined the effects of gestational exposure to second hand smoke on alcoholic liver injury in adult male C57BL/6 mice. Pregnant mice were exposed to air or second-hand smoke for 4 h/day from gestational day 6–19. Pups were culled to 6 pups/litter with litters of equal average weight and fed chow diets ad libitum until post-natal day 65. At that time groups of *n* = 20 male pups either continued on chow or were switched to high fat Lieber DeCarli liquid diets and were fed ethanol up to a final concentration of 28% total calories or pair-fed diets in which the ethanol calories were matched by dextrose-maltodextrin for 16 weeks. As shown in Figure 2, no effects of gestational second-hand smoke on adult body weight or liver pathology were observed in any diet group. However, the pair-fed “controls” had significantly increased body weight >30%, increased % liver weight, increased liver triglycerides and had dramatically increased serum alanine aminotransferase (ALT) values (*p* < 0.05) indicative of development of non-alcoholic steatohepatitis (NASH). In contrast, the ethanol-fed group had lower weights than the pair-fed mice, consistent with reports that ethanol is treated as “empty calories” [13] and had smaller increases in % liver weight and serum ALTs relative to chow-fed mice, despite having higher levels of hepatic triglycerides than pair-fed mice (*p* < 0.05). Development of obesity and fatty liver pathology in the pair-fed controls makes interpretation of any alcohol effects in such studies very difficult.

Feeding of ethanol intragastrically to rats with diets high in polyunsaturated fat where carbohydrate calories are limited resulted in development of steatohepatitis and an increase in necroinflammatory injury relative to high fat controls [25,28]. Under these conditions, the increase in pathology appears to be associated with increased induction of the ethanol-inducible enzyme cytochrome P450 CYP2E1, which has been characterized as an important source of reactive oxygen species (ROS) [26,28]. In this model we have demonstrated that liver pathology including steatosis disappears when polyunsaturated fats are substituted with a mixture of medium and long chain saturated fats, even when dietary ethanol content is the same [13]. Reduction in liver pathology in rats fed this diet appeared to be due to a reduction in susceptibility to ROS-mediated membrane peroxidation and increases in peroxisome proliferator activated receptor alpha (PPARα)-mediated fatty acid oxidation [13]. More recent studies with double knockout mice lacking the enzymes, glutathione *S*-transferase A4-4 and PPARα, suggest that lipid peroxidation products such as 4-hydroxy-2-nonenal (4-HNE) derived from free radical degradation of polyunsaturated fatty acids are important in the progression of liver pathology beyond simple steatosis. The double knockout mice had increased 4-HNE protein adducts, higher serum ALT, increased production of inflammatory cytokines, including tumor necrosis factor alpha (TNF-α) and interferon gamma (IFN-γ), increased evidence of matrix remodeling, and more fibrosis than ethanol-fed wild type or single knockout mice [29]. 4-HNE adduction of hepatic proteins identified by immunohistochemistry and LC-MS/MS proteomics analysis, particularly in the mitochondria, appear to result in metabolic dysfunction including defects in fatty acid homeostasis and ammonia metabolism [30]. These protein adducts also appear to act as haptens stimulating autoimmune responses which may contribute to the development of necroinflammatory responses and progression of liver injury [29]. Interestingly, we have established a similar role for lipid peroxidation of polyunsaturated fatty acids in the progression of non-alcoholic fatty liver disease in both rat intragastric feeding and in GSTA4-4/PPARα double knockout mice models [31,32]. 

### 2.3. Betaine: A Promising Therapeutic in the Treatment of Alcohol-Induced Liver Injury

Kusum K. Kharbanda

Research conducted over past several decades has demonstrated that chronic alcohol exposure causes liver damage by several mechanisms, as recently reviewed [33,34,35,36]. It is well-established that the pathogenesis of alcoholic liver disease involves not only multiple factors but also involves cross-talk among multiple organs/tissues, the most notable being the white adipose tissue (WAT)-liver [37,38,39,40] and the gut-liver [41,42,43,44,45,46] axes. Our laboratory and others have made seminal contributions in elucidating how ethanol exposure alters the methionine metabolic pathway in the liver [47,48,49,50,51,52]. Later investigations, largely conducted in my laboratory, revealed that these alterations are also seen in WAT and the various gastrointestinal segments following ethanol exposure. Interestingly, this unifying mechanism not only helps to understand how ethanol can adversely affect multiple organs of interest, but also provides a singular therapeutic approach for mitigating ethanol effects on the liver, WAT, gut, and other organs. Briefly, we have shown that ethanol exposure predominantly inhibits the activities of methionine synthase and methionine adenosyltransferase, two vital cellular enzymes involved in remethylating homocysteine and generating *S*-adenosylmethionine (SAM), respectively. To compensate for these losses, ethanol feeding increases the activity of betaine-homocysteine methyltransferase (BHMT) that utilizes betaine to remethylate homocysteine and maintain normal levels of SAM. However, during extended periods of ethanol feeding, the alternate homocysteine remethylation pathway cannot be maintained because of a depletion of endogenous betaine stores. This results in decreasing the SAM levels while increasing the levels of two toxic metabolites, homocysteine and *S*-adenosylhomocysteine (SAH) and the subsequent reduction in the cellular SAM:SAH ratio [5,49,50,51,52]. The ethanol-induced reduction in the ratio in the liver, WAT and the various intestinal segments produces diverse, but profound, functional consequences in these three organs/tissues as detailed below. This occurs because SAM:SAH ratio is an important metabolic indicator for cellular methylation status and regulates the activity of many of the 120 members of SAM-dependent methyltransferases. It has been shown that decreasing the SAM:SAH ratio correspondingly impairs the activity of several methyltransferases [53]. This occurs because SAH has a high affinity binding to the catalytic region of many methyltransferases, especially for those with a lower K**_i_**value for SAH than the K_m_ value for SAM [53]. Since every organ/tissue has a repertoire of specific methyltransferases that play critical roles in maintaining their functional homeostasis, a reduction in the cellular SAM:SAH ratio generates specific consequences in each of these and other organs/tissues. These organ/tissue-specific consequences, in turn, modulate the gut-liver and liver-WAT axes producing adverse events that ultimately culminate in progressive liver injury. 

*Functional Consequences of Lower SAM:SAH Ratio in the Liver.* Five important liver SAM-dependent methyltransferases are: phosphatidylethanolamine methyltransferase, isoprenylcysteine carboxyl methyltransferase, protein-isoaspartate methyltransferase, guanidinoacetate methyltransferase, and protein arginine methyltransferase. Focusing on these methyltransferases, we reported that alcohol-induced reduction in the hepatocellular SAM:SAH ratio specifically impairs the reactions catalyzed by these enzymes, thereby decreasing methylation of their respective targets [5,54,55,56,57,58]. This results in decreased secretion of very-low density lipoproteins, impaired activation of GTPases, diminished protein repair, reduced creatine biosynthesis and arginine methylation, respectively [5,56,57,58,59]. Ultimately, these defects contribute to the development of steatosis [5], increased apoptosis [56], accumulation of damaged proteins [57,60], reduction in creatine levels [58], and altered signaling [61] –all of which are hallmark features of early alcoholic liver injury. 

*Functional Consequence of Lower SAM:SAH Ratio in the WAT.* We have shown that alcohol-induced reduction in WAT SAM:SAH ratio through a methylation-dependent pathway activates two main lipases (hormone-sensitive lipase and adipose triglyceride lipase) which enhance WAT lipolysis [38,39]. The consequent increased circulating non-esterified free fatty acids are transported to the liver and become deposited there as triglycerides, contributing to the development of hepatic steatosis. Further studies have revealed that the alcohol-induced increased WAT homocysteine levels cause a reduction in adiponectin production and secretion [37].

*Functional Consequence of Lower SAM:SAH Ratio in the Gut*. Using both in vivo and in vitro approaches [62], we have shown that a reduction in SAM:SAH ratio through a methylation-dependent process caused tight junction disruption, as evident from the disorganized localization of three key members (occludin, claudin-1 and ZO-1) of the multiprotein tight junction complex. This loss of barrier integrity ultimately causes portal circulation endotoxemia, a necessary etiological factor that promotes liver inflammation.

*Betaine Treatment.* We further showed that treatment with betaine, a nutrient in itself and a metabolite product of choline, lowers cellular SAH to maintain normal SAM:SAH ratio [5,54]. This preserves the essential methylation reactions in the liver, WAT and the gut, which thereby prevents the development of alcoholic organ injury, especially progressive liver damage [5,51,52,57,59]. 

### 2.4. Malnutrition and Nutrition Support in Alcoholic Liver Disease: Clinical Relevance

Samir Zakhari and Craig J. McClain 

The liver is the largest and possibly the most metabolically complex organ in the body. The liver plays a vital role in protein, carbohydrate, and fat metabolism, as well as the metabolism of important micronutrients. With the development of alcoholic liver disease (ALD) in patients, there is frequently an associated altered nutritional status, especially with advanced ALD. A widely recognized phenotype for malnutrition in severe alcoholic hepatitis (AH)/advanced ALD is skeletal muscle loss (sarcopenia) with or without loss of fat mass [63,64].

**Figure 3:**
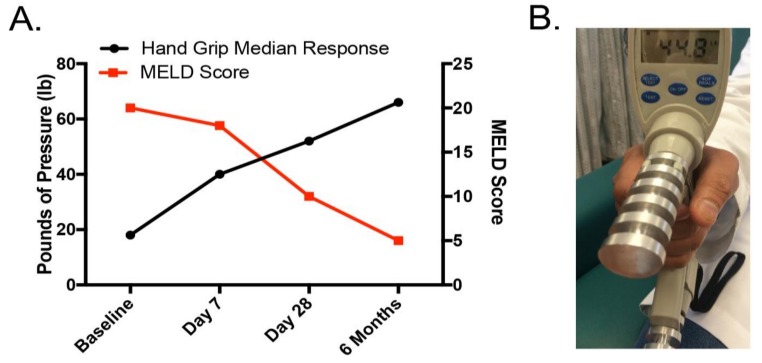
Time-course handgrip assessment in patient with moderate alcoholic hepatitis. (**A**) Correlation analysis between hand grip response and Model for End-Stage Liver Disease (MELD) score; (**B**) hand grip dynamometry.

It is important to do an initial assessment of nutritional status and to perform systematic nutritional follow-up examinations in patients with alcoholic hepatitis/alcoholic cirrhosis. Unfortunately, there are no gold standard techniques or widely-accepted strategies for assessment of malnutrition in liver disease in the clinical setting [63]. Moreover, underlying liver disease itself impacts many of our standard tests of malnutrition. Ten potential mechanisms of nutritional assessment are listed in Table 1. Anthropometry measurements, such as triceps skinfold or body mass index (BMI), are widely used but can be impacted by fluid retention that is frequently seen in cirrhosis [65,66]. Biological parameters, such as visceral protein, are impacted by the fact that visceral proteins are made in the liver, and liver disease can decrease visceral protein production [67]. Subjective global assessment (SGA) is a simple, bedside evaluation of nutritional status that we regularly utilize in patients with ALD [68,69]. The SGA includes patient history regarding weight loss, usual dietary intake, functional capacity, gastrointestinal symptoms, and evidence of malnutrition on physical exam (loss of muscle or fat mass or presence of edema) [68]. Using this information, patients are classified as: (i) well nourished; (ii) moderately malnourished; or (iii) severely malnourished. Bioelectrical impedance (BIA) technology has recently improved, and this technique is increasingly used in liver disease [66]. BIA involves introducing a small electric current through the body. Each body tissue has a specific electrical conductivity which is directly related to the water and electrolyte content of that tissue. Pirlich and coworkers showed a strong correlation between BIA and the gold standard of total body potassium for assessing malnutrition [70]. They concluded that BIA provided reliable estimates of body cell mass, even in patients with ascites, and it was superior to other bedside techniques. We regularly evaluate handgrip strength by handgrip dynamometry, an easily performed, inexpensive and noninvasive technique. Handgrip strength has been correlated with other markers of malnutrition in liver disease. It provides an important indicator of functional status, and it can improve with nutritional supplementation [66,71]. This test is especially useful for monitoring patient progress over time, as shown in Figure 3. Probably the most detailed reports describing malnutrition in alcoholic hepatitis (AH) are from large studies from the Veterans Health Administration (VA) Cooperative Studies Program [10,72,73,74]. It has been demonstrated that virtually every patient with AH had some degree of malnutrition [10]. Patients had a mean alcohol consumption of 228 g/day (>15 drinks/day with approximately 50% of energy intake from alcohol). Thus, while calorie intake was generally adequate, there was inadequate intake of protein and critical micronutrients. Similar data were observed in a follow-up VA study on alcoholic hepatitis [75].

A research interest of our group has been whether nutritional abnormalities occur before the development of alcohol-induced liver injury (potentially contributing to the liver injury) or whether nutritional alterations occur only subsequent to liver injury. There are compelling animal data showing that nutritional deficiencies (such as zinc deficiency) occur early in experimental ALD in mice/rats, and that nutritional deficiencies contribute to the development/progression of liver injury/inflammation [43,76]. Indeed, other presentations in this symposium highlight the importance of nutritional factors such as dietary fat in the development of experimental ALD. There are also compelling human data showing that when liver disease becomes more advanced, malnutrition frequently occurs. This is true whatever the etiology of liver disease (alcohol, viral or metabolic). We recently evaluated a cohort of 48 otherwise healthy participants with alcohol use disorder but no clinical signs of ALD and no clinical evidence of malnutrition who were participating in a treatment program. They averaged 15 drinks per day, and an average of 15 years of heavy drinking [77]. Thus, these chronic alcoholics without clinical liver disease had alcohol consumption levels very similar to those observed in VA cooperative studies and other clinical trials of severe alcoholic hepatitis/alcoholic cirrhosis. These chronic alcoholics with no overt clinical evidence of malnutrition were consuming over 1500 calories a day of “empty calories.” However, more detailed investigations revealed that these chronic alcoholics often had subtle individual nutrient deficiencies, such as zinc deficiency. Moreover, those subjects with zinc deficiency were more likely to have modest elevations in their liver enzymes that were indicative of early alcohol-induced liver injury. Thus, our human data are consistent with animal studies which suggest that altered nutrition occurs early in the development of ALD and may play a mechanistic role in the development of liver disease.

Several studies of hospitalized ALD patients suggest that enteral nutritional support improves nutritional status and may improve outcome. A relatively high protein intake is usually recommended (1.2–1.5 g/kg body weight (BW)/day) [63,64]. It is important to monitor food intake because of the high risk for malnutrition and the frequency with which feeding is interrupted in the hospital. In subjects with inadequate oral intake, early enteral nutrition is suggested. Similarly, a low-sodium product that is calorically dense should be used. Placing a feeding tube has not been shown to increase the risk of bleeding from esophageal varices, and a high-protein diet does not increase the risk of encephalopathy [63,64]. On discharge, patients should continue to receive dietary counseling and nutritional assessment and support. Patients with ALD and cirrhosis should receive a late-night snack (9.00 p.m.) to decrease an overnight starvation state which contributes to muscle breakdown.

In summary, patients with ALD, especially those with advanced cirrhosis/alcoholic hepatitis, frequently have malnutrition that can impact outcome. Nutritional assessment and appropriate nutritional therapy can have a positive impact on nutritional status and quality of life and may improve survival.

## Figures and Tables

**Table 1:** Methods of Assessment of Malnutrition.

	Methods
**1**	Anthropometry
**2**	Biologic Indicators
**3**	Creatinine Height Index
**4**	Muscle Strength
**5**	Bioelectrical Impedance
**6**	Air Displacement, Plethysmography
**7**	Imaging (DEXA, MRI, CT, etc.)
**8**	Subjective Global Assessment
**9**	Energy Balance
**10**	Metabolomics

DEXA: dual-energy X-ray absorptiometry; MRI: magnetic resonance imaging; CT: computed tomography scan.

## Abbreviations

AA	arachidonic acid
ALD	alcoholic liver disease
AH	alcoholic hepatitis
BIA	bioelectrical impedance
BHMT	betaine-homocysteine methyltransferase
BMI	body mass index
ChREBP	carbohydrate response element binding protein
HETEs	hydroxyeicosatetraenoic acids
HODEs	hydroxyoctadecadienoic acids
LA	linoleic acid
NASH	non-alcoholic steatohepatitis
4-HNE	4-hydroxynonenal
OXLAMs	oxidized linoleic acid metabolites
PUFA	polyunsaturated fatty acid
ROS	reactive oxygen species
SAH	*S*-adenosylhomocysteine
SAM	*S*-adenosylmethionine
SREBP-1c	steroid regulatory element binding protein 1c
SGA	Subjective global assessment
WAT	white adipose tissue
USF	unsaturated fat
